# Contribution of Dietary Supplements to Nutritional Adequacy by Socioeconomic Subgroups in Adults of the United States

**DOI:** 10.3390/nu10010004

**Published:** 2017-12-22

**Authors:** Jeffrey B. Blumberg, Balz Frei, Victor L. Fulgoni, Connie M. Weaver, Steven H. Zeisel

**Affiliations:** 1 Antioxidants Research Laboratory, Jean Mayer USDA Human Nutrition Research Center on Aging, and the Friedman School of Nutrition Science and Policy, Tufts University, Boston, MA 02155, USA; jeffrey.blumberg@tufts.edu; 2 Linus Pauling Institute and Department of Biochemistry & Biophysics, Oregon State University, Corvallis, OR 97331, USA; balz.frei@oregonstate.edu; 3 Nutrition Impact, LLC, Battle Creek, MI 49014, USA; 4 Department of Nutrition Science, Purdue University, West Lafayette, IN 47907, USA; weavercm@purdue.edu; 5 Nutrition Research Institute, Department of Nutrition, University of North Carolina, Kannapolis, NC 28081, USA; steven_zeisel@unc.edu

**Keywords:** vitamin/mineral supplement, NHANES, micronutrients, Poverty Income Ratio (PIR)

## Abstract

Many Americans have inadequate intakes of several nutrients, and the Dietary Guidelines for Americans 2015–2020 identified vitamins A, C, D, and E, in addition to calcium, magnesium, iron, potassium, choline, and fiber as “underconsumed nutrients”. Based on nationally representative data on 10,698 adults from National Health and Nutrition Examination Surveys (NHANES), 2009–2012, assessments were made of socioeconomic differences, based on the Poverty Income Ratio (PIR), in terms of the association of dietary supplement use on nutrient intake and nutrient inadequacies. Compared to food alone, the use of any dietary supplement plus food was associated with significantly (*p* < 0.01) higher intakes of 15–16 of 19 nutrients examined in all socioeconomic groups; and significantly reduced rates of inadequacy for 10/17 nutrients in the subgroup PIR > 1.85 (not poor), but only 4–5/17 nutrients (calcium and vitamins A, C, D, E) for the poor and nearly poor subgroups (PIR < 1.35 and PIR 1.35 to ≤1.85, respectively). An increased prevalence of intakes above the Tolerable Upper Intake Level (UL) was seen for 3–9/13 nutrients, but all were less than 5% in the PIR subgroups. In conclusion, dietary supplement use was associated with an increased micronutrient intake, decreased inadequacies, and a slight increase in the prevalence of intakes above the UL, with greater benefits seen in the PIR > 1.85 subgroup.

## 1. Introduction

Micronutrients are required for nearly all metabolic and developmental/growth processes, and adequate intakes are needed for overall health, growth and development, healthy aging, and well-being across the lifespan. The Dietary Guidelines for Americans 2015–2020 (DGA) recommends consuming nutrient-dense foods as part of a healthy eating pattern and, in some cases, fortified foods and dietary supplements to help achieve and maintain a healthy body weight, support nutrient adequacy, and reduce the risk of chronic disease [1]. In spite of repeated recommendations from Dietary Guidelines, many Americans have inadequate intakes of several essential nutrients [1]. The DGA identified vitamin A, vitamin C, vitamin D, vitamin E, choline, calcium, iron (for certain age/gender groups), magnesium, potassium, and fiber as “underconsumed nutrients”, and vitamin D, calcium, iron, potassium, and fiber as “nutrients of public health concern” because low intakes are associated with a risk for the development of chronic disease [1].

The Second National Report on Biochemical Indicators of Diet and Nutrition in the U.S. Population reported that nutrition deficiencies varied by age, gender, or race/ethnicity and could be as high as nearly one third of certain population groups [2]. Socioeconomic status (SES) is now being routinely included in dietary studies as an independent variable in the analysis of nutrition and health status [3]. It is generally accepted that dietary choices and nutrient intakes are influenced by SES, and population subgroups in low SES are more likely to have a poorer quality of dietary intake than those in high SES [4,5,6,7,8]. A number of studies have also reported that healthier diets cost more than unhealthy diets, and diet cost is an important predictor of the quality of diet and nutrient intake [9,10,11]. Family income was also an independent contributor to the differences in nutrient intake patterns between low- and high-SES households [12,13].

The use of dietary supplements is mostly a health and lifestyle choice, and the key motivators for consumers are maintenance or improvement in overall health, as well as to obtain specific health benefits [14,15]. Dietary supplement consumption has increased over time in the United States [16]; currently, about 50% of adults take dietary supplements and more than two-thirds of them use vitamin/mineral supplements [17,18,19]. The consumption of dietary supplements has been shown to increase overall nutrient intake and decrease the prevalence of nutrient inadequacy [20].

A few studies have examined the effect of socioeconomic differences on the consumption of dietary supplements and nutrient intake, with most [21,22,23], but not all [24], indicating that dietary supplement use was higher in those with higher incomes. Additionally, USDA has released the intake from food and dietary supplements by PIR subgroups, but did not assess usual intakes or nutrient adequacy [25]. The primary objective of this cross-sectional study was to assess the association of dietary supplement use with usual nutrient intakes and nutrient adequacy in three distinct SES subgroups using a large nationally representative data set. This study was part of a broader effort to determine the association of dietary supplement use on nutrient intake and nutrient adequacy among diverse US populations [26].

## 2. Materials and Methods

### 2.1. Study Population

We used the data from National Health and Nutrition Examination Survey (NHANES) surveys examining a representative sample of the civilian, non-institutionalized US population. The present analysis combined two NHANES datasets: 2009–2010 and 2011–2012 for adults aged 19 years and older with reliable 24-h recall dietary interviews (*n* = 10,698). Pregnant and/or lactating females and those with incomplete or unreliable 24-h recall data were excluded. All participants provided written informed consent and the Research Ethics Review Board at the NCHS approved the survey protocol [27]. Participants were categorized into income groups based on the Poverty Income Ratio (PIR), which was determined by dividing family income by the poverty threshold specific to a family size and geographic location [28]. The combined sample included 3589 adults at PIR < 1.35 (poor), 1184 adults at PIR 1.35–1.85 (nearly poor), and 5012 adults at PIR > 1.85 (not poor). These groups were selected as the two lower levels are used to determine eligibility for various federal programs (e.g., Head Start, Supplemental Nutrition Assistance Program (SNAP), Special Supplemental Nutrition Program for Women, Infants, and Children (WIC), etc.).

### 2.2. Micronutrient Intake from Foods

Dietary intake data from two reliable 24-h dietary recall interviews using United States Department of Agriculture’s (USDA) automated multiple-pass method (AMPM) were used [24]. The Food and Nutrient Database for Dietary Studies (FNDDS) 2009–2010 and 2011–2012 [29,30] (based on the USDA National Nutrient Database for Standard Reference (SR) releases 24 and 26 [31], respectively) were used to determine the nutrient content of foods consumed by the NHANES participants. 

### 2.3. Micronutrient Intake from Supplements

A dietary supplement questionnaire assessing the usage of vitamins, minerals, botanicals, and other dietary supplements over the past 30 days was administered as part of the NHANES household interview [32]. The average daily intake of nutrients from dietary supplements was calculated for individuals based on the supplement consumption frequency and dosage. 

### 2.4. Statistics

Usual intake including distribution and percentages meeting cutoffs was estimated using version 2.1 of the National Cancer Institute (NCI) usual intake SAS macro programs [33]. The estimates were generated using two days of dietary data with the use of day 1 dietary weights in all stages of the estimation process. Balanced repeated replication (BRR) with a Fay adjustment factor of 0.3 was used for variance estimates. Covariates included Dietary Reference Intake age group, day of recall, and weekday/weekend flag and a supplement usage flag (yes/no). Supplement intake (30-day supplement intake), was directly added to usual intake from food alone to obtain the estimated usual intake for diet plus supplements. The cut-point method of estimating percent less than a cutoff was used in all cases except for iron. For iron, published risk curves and numerical integration were used to produce estimates using the probability method. Percentage of the population below the Estimated Average Requirement (EAR) for 17 nutrients (calcium, copper, iron, magnesium, phosphorus, selenium, zinc, vitamin A, thiamin, riboflavin, niacin, folate vitamin B6, vitamin B12, vitamin C, vitamin D, and vitamin E) was determined. In addition, the percentage of the population above the Adequate Intake (AI) for two nutrients (vitamin K and choline; given an EAR has not been established the percentage of the population with inadequate intakes cannot be determined [34]), and above the Upper Tolerable Level (UL) for 13 nutrients (calcium, copper, iron, phosphorus, selenium, zinc, vitamin A as retinol, folate as folic acid, vitamin B6, vitamin C, vitamin D, vitamin E as added alpha-tocopherol, and choline) was also assessed [34,35]. Potassium and sodium were not included in the present analysis as negligible amounts are found in dietary supplements. NHANES survey weights, strata, and primary sampling units were used in all calculations, thus providing nationally representative estimates (for NHANES response rates see [36]). A Z-statistic was used to test whether means and proportions of the population below EAR or above the AI or UL were similar across socioeconomic groups; *p* < 0.01 was deemed significant. Data are presented as mean ± SE. All statistical analyses were performed with SAS software (version 9.2; SAS Institute Inc., Cary, NC, USA) and SUDAAN (version 11; Research Triangle Institute; Raleigh, NC, USA).

### 2.5. Trial Registration 

Not applicable; as this is secondary analysis of publicly released observational data (NHANES 2009–2012)

## 3. Results

### 3.1. General Demographics and Dietary Supplement Usage

The age (mean ± SE) of the subjects was 42.2 ± 1.5, 46.1 ± 0.9, and 48.7 ± 0.5 years in the PIR < 1.35, PIR 1.35–1.85, and PIR < 1.35 subgroups, respectively, while the percentage of each group being female was 53.6 ± 1.1, 51.1 ± 1.8, and 49.3 ± 0.8%, respectively. The percentage of non-Hispanic White, non-Hispanic Black, and Mexican Americans in the subgroups were 50.8 ± 4.0, 17.7 ± 2.6, and 14.9 ± 2.6% for the PIR < 1.35 subgroup; 54.0 ± 4.1, 18.0 ± 2.2, and 12.3 ± 2.4% for the PIR 1.35–1.85; and 78.1 ± 41.9, 7.7 ± 1.9, and 4.3 ± 0.7% for the PIR < 1.35 subgroups, respectively. Dietary supplement use was reported by 39.2 ± 1.7% of adults in the PIR < 1.35 subgroup, 50.0 ± 2.5% of adults in the PIR 1.35–1.85 subgroup, and 62.2 ± 0.9% adults in PIR > 1.85 subgroup of NHANES 2009–2012 participants.

### 3.2. Comparison of Intakes from Food Alone in Dietary Supplement Consumers and Non-Consumers

Adult consumers of dietary supplements in the PIR < 1.35 subgroup had higher (*p* < 0.01) intakes of vitamin A from food alone, but lower intakes of selenium, niacin, and choline compared to non-consumers (Table 1). Consumers of dietary supplements in the PIR 1.35–1.85 subgroup had higher intakes of vitamins A, E, and K from food alone, while consumers in the PIR > 1.85 subgroup had higher intakes of copper, magnesium, riboflavin, and vitamins A, C, E and K from food alone compared to respective non-consumers. Regarding the percentage of the population with inadequate intakes from food alone (Table 2), consumers of dietary supplements in the PIR < 1.35 subgroup had a lower inadequacy for vitamin A than non-consumers, while those in the PIR 1.35–1.85 subgroup had a lower inadequacy for vitamin A and a greater percentage of the population exceeding the AI for vitamin K compared to non-consumers. Consumers of dietary supplements in the PIR > 1.85 subgroup had a lower inadequacy for magnesium and vitamins A and C, and a greater percentage of the population exceeding the AI for vitamin K from food alone compared to non-consumers. Both dietary supplement consumers and non-consumers had high percentages of the population below the EAR from food only for calcium, magnesium, and vitamins A, C, D, and E, along with relatively low percentages of the population with intakes above the AI for vitamin K and choline.

### 3.3. Effect of Supplement Use on Usual Intake of Nutrients

The usual intake of nutrients from food and supplements combined was significantly higher (*p* < 0.01) than from food only for all nutrients except phosphorus, vitamin K, and choline in all PIR subgroups (Table 3). However, the difference in magnesium was only significant for those in the PIR > 1.85 subgroup. Usual intakes of DGA-identified “underconsumed nutrients” increased (range of percentage increase of mean intakes) significantly by 9–19% for calcium, 15–21% for iron, 32–56% for vitamin A, 47–115% for vitamin C, 95–232% for vitamin D, and 145–227% for vitamin E in the various PIR subgroups. The magnitude of increased intake was generally higher for high-income than for low-income population subgroups. 

### 3.4. Effect of Supplement Use on Prevalence of Inadequacy

The consumption of dietary supplements was associated with a decreased (*p* < 0.01) prevalence of inadequacy (intakes below EAR) for DGA-identified “underconsumed nutrients” by (range of percentage decrease of inadequacy) 17–34% for calcium, 15–29% for vitamin A, 19–39% for vitamin D, and 18–36% for vitamin E across all socioeconomic subgroups (Table 4). The prevalence of inadequacy of vitamin C also decreased by 18–32% associated with supplement intake; however, the decrease was only significant in “not poor” (PIR > 1.85) and “poor” (PIR < 1.35) population subgroups, and not significant in the “nearly poor” subgroup (PIR 1.35–1.85). There was also a decrease (*p* < 0.01) in % population with intakes below EAR for iron, magnesium, zinc, folate, and vitamin B6 associated with supplement intake in the PIR > 1.85 subgroup only. There were no statistically significant differences in % population with intakes above AI of vitamin K or choline associated with supplement intake (plus food) compared to food only among adults in any socioeconomic subgroup.

### 3.5. Comparison of Prevalence of Inadequacy by PIR

**Figure 1:**
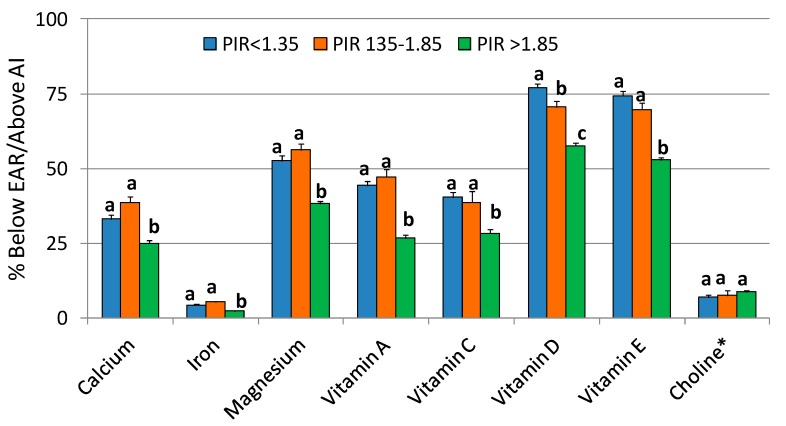
Comparison of percent adult (19+ years old) population below Estimated Average Requirement (EAR) or above Adequate Intake (AI) of DGA-identified “underconsumed nutrients” from foods + dietary supplements among different socioeconomic subgroups. NHANES 2009–2012 gender combined data. Bars with different letters for one nutrient are significantly different at *p* < 0.01. * AI Nutrient.

There were significant differences by SES subgroup in the proportion of the population with intakes (from food + supplement) below EAR (Table 4, Figure 1). The “not poor” (PIR > 1.85) subgroup had a lower prevalence of inadequacy for most nutrients compared to the other two PIR subgroups, including DGA-identified “underconsumed nutrients”: (range of differences in inadequacy) 25–36% for calcium, 48–58% for iron, 27–32% for magnesium, 40–43% for vitamin A, 27–30% for vitamin C, 18–25% for vitamin D, and 24–29% for vitamin E. The PIR > 1.85 population subgroup also had a higher proportion of the population with intakes of vitamin K (from food + supplement) above the AI.

### 3.6. Effect of Supplement Use on Prevalence of Intakes above the UL

There was a higher (*p* < 0.01) prevalence of intakes above UL for calcium (PIR < 1.35 and PIR > 1.85), iron (all PIR subgroups), selenium (PIR > 1.85), zinc (all PIR subgroups), vitamin A (PIR > 1.85), folate (all PIR subgroups), vitamin B6 (PIR < 1.35 and PIR > 1.85), vitamin C (PIR > 1.85), and vitamin D (PIR < 1.35 and PIR > 1.85) associated with dietary supplement consumption (Table 5). However, the actual percentages above the UL were mostly below 2% and were never more than 5% for any nutrient and SES subgroup.

## 4. Discussion

This NHANES (2009–2012) analysis indicates that dietary supplement use was associated with a reduction of a range of micronutrient inadequacies among US adults of diverse SES. 

The prevalence of inadequate micronutrient intake was dependent on SES. Adults with poor SES (PIR < 1.35) had a higher prevalence of micronutrient inadequacies, including the DGA-identified “underconsumed nutrients” and “nutrients of public health concern”, compared to adults with higher SES (PIR > 1.85). High SES was previously reported to be associated with healthier and nutrient-dense dietary patterns and a higher diet quality [4,5,6,7,8], and diet cost was hypothesized to be an important predictor of dietary quality [9,10,11]. However, only a few studies have investigated the association between socioeconomic status and vitamin/mineral supplement use. We found a higher supplement use among those in a higher income category compared to those in a lower income category. Previous analyses of NHANES also indicate a positive association between supplement use and income level [37,38], with higher-income adults more likely to take calcium supplements than those with lower incomes [39]. Household income has also been shown to be related to supplement use; people in the highest income households were 60% more likely to take supplements than those in the lowest income households in a Canadian study [23]. While there was generally an increased micronutrient intake associated with dietary supplements across all socioeconomic groups, the decreases in prevalence of inadequacy among the PIR < 1.35 and PIR 1.35–1.85 subgroups were not as large as in the PIR > 1.85 subgroup. The poorer subgroups had lower intakes of some nutrients from food alone and the lower percentage of those populations taking dietary supplements also influences these results. 

Calcium, potassium, iron (adolescent and adult females), magnesium, dietary fiber, choline, and vitamins A, C, D, and E are identified as “underconsumed nutrients”, and vitamin D, calcium, iron, potassium, and fiber as “nutrients of public health concern” [1]. Nutrient deficiencies have been associated with an increased risk of several adverse health effects including cardiovascular disease, stroke, impaired cognitive function, cancer, eye diseases, poor bone health, and other conditions [2,40,41,42]. Low intakes of calcium, potassium, iron (adult females) dietary fiber, and vitamin D are associated with health concerns [1]. The use of supplements was associated with significantly increased nutrient intakes and decreased the percent population with inadequate intakes (not meeting the EAR) for most nutrients in all socioeconomic population subgroups.

Given that our results indicate lower intakes from food alone in the lower two PIR subgroups, even though at least some in the lower income subgroups were likely receiving support from existing food assistance programs, some consideration should be given as to whether dietary supplements should be subsidized or given to those eligible for food assistance. Additional research should be undertaken to isolate those already receiving food assistance and assessing whether dietary supplements could further improve nutrient adequacy.

The use of a large nationally representative population-based sample of adults with the most recent available data was a major strength of our study. One of the limitations was that the estimates of nutrient intakes relied on self-reported data and as such were subject to bias. However, 85% of the time, the NHANES interviewers examined dietary supplement bottles/labels and verified the self-reporting accuracy [17]. Our analysis relied on the assumption that 24-h dietary recalls-based nutrient intakes from food sources were unbiased and self-reported dietary supplement intake accurately reflected long-term supplement intake patterns. Furthermore, estimates of vitamins and minerals contributed by dietary supplements depended on the label declarations rather than analytic values. 

In conclusion, the results of this study reveal an association between supplement use and meeting nutritional adequacies for various nutrients in all SES groups, and that the impact is larger for higher rather than lower socioeconomic population subgroups. 

## Figures and Tables

**Table 1:** Usual intake of nutrients from foods only among adults (19+ years old) by PIR groups. NHANES 2009–2012, gender combined data.

Nutrients	PIR < 1.35		1.35 ≤ PIR ≤ 1.85		PIR > 1.85	
	Non-Consumer (*n* = 2165)	Consumer (*n* = 1424)	Non-Consumer (*n* = 635)	Consumer (*n* = 549)	Non-Consumer (*n* = 2026)	Consumer (*n* = 2986)
Nutrients with EAR						
Calcium (mg)	1003 ± 22	992 ± 28	915 ± 31	957 ± 31	1005 ± 18	1040 ± 14
Copper (mg)	1.21 ± 0.02	1.23 ± 0.02	1.14 ± 0.04	1.29 ± 0.05	1.31 ± 0.02	1.42 ± 0.03 *
Iron (mg)	15.2 ± 0.2	15.5 ± 0.3	14.0 ± 0.4	14.8 ± 0.5	15.6 ± 0.3	16.0 ± 0.2
Magnesium (mg)	291 ± 5	297 ± 5	275 ± 7	296 ± 10	309 ± 4	331 ± 4 *
Phosphorus (mg)	1423 ± 21	1359 ± 29	1312 ± 32	1347 ± 34	1450 ± 18	1447 ± 14
Selenium (µg)	117 ± 2	109 ± 2 *	110 ± 3	107 ± 3	118 ± 2	114 ± 1
Zinc (mg)	11.8 ± 0.2	11.2 ± 0.2	10.9 ± 0.4	10.9 ± 0.3	12.0 ± 0.2	12.1 ± 0.2
Vitamin A (µg RE)	546 ± 12	636 ± 28 *	484 ± 23	621 ± 26 *	623 ± 15	742 ± 24 *
Thiamin (mg)	1.62 ± 0.03	1.64 ± 0.03	1.54 ± 0.04	1.55 ± 0.04	1.68 ± 0.02	1.70 ± 0.02
Riboflavin (mg)	2.03 ± 0.04	2.11 ± 0.06	1.89 ± 0.05	2.08 ± 0.07	2.19 ± 0.03	2.30 ± 0.03 *
Niacin (mg)	26.8 ± 0.6	24.6 ± 0.5 *	24.6 ± 0.7	23.9 ± 06	27.1 ± 0.4	26.4 ± 0.3
Folate (µg DFE)	539 ± 11	548 ± 12	1.93 ± 0.06	523 ± 20	565 ± 10	582 ± 9
Vitamin B_6_ (mg)	2.11 ± 0.07	2.04 ± 0.05	2.07 ± 0.07	1.92 ± 0.06	2.18 ± 0.05	2.21 ± 0.03
Vitamin B_12_ (µg)	5.23 ± 0.15	5.08 ± 0.02	4.91 ± 0.27	4.72 ± 0.24	5.40 ± 0.13	5.60 ± 0.12
Vitamin C (mg)	81.6 ± 3.9	80.5 ± 4.2	78.4 ± 7.0	88.2 ± 7.8	76.0 ± 2.4	94.2 ± 2.6 *
Vitamin D (µg)	4.76 ± 0.15	4.97 ± 0.30	4.18 ± 0.20	4.43 ± 0.21	4.81 ± 0.15	5.00 ± 0.14
Vitamin E (mg)	7.77 ± 0.21	7.74 ± 0.14	6.75 ± 0.23	8.18 ± 0.33 *	8.49 ± 0.18	9.34 ± 0.17 *
Nutrients with AI						
Vitamin K (µg)	86.0 ± 3.5	90.8 ± 4.8	78.9 ± 4.1	105 ± 7 *	102 ± 3	127 ± 5 *
Choline (mg)	341 ± 7	313 ± 7 *	317 ± 9	323 ± 11	349 ± 6	342 ± 4

* Significant difference for consumer and non-consumer within PIR subgroups at *p* < 0.01.

**Table 2:** Percent of adult (19+ years old) population below Estimated Average Requirement (EAR) or above Adequate Intake (AI) of nutrients from foods only by PIR subgroups. NHANES 2009–2012 gender combined data.

Nutrients	PIR < 1.35		1.35 ≤ PIR ≤ 1.85		PIR > 1.85	
	Non-Consumer (*n* = 2165)	Consumer (*n* = 1424)	Non-Consumer (*n* = 635)	Consumer (*n* = 549)	Non-Consumer (*n* = 2026)	Consumer (*n* = 2986)
Nutrients with EAR, percentage below EAR						
Calcium	36.3 ± 2.1	44.2 ± 2.2	47.1 ± 3.5	49.6 ± 3.0	36.1 ± 1.7	38.2 ± 1.4
Copper	8.3 ± 1.2	5.9 ± 1.2	9.8 ± 2.9	7.6 ± 2.0	4.6 ± 0.8	1.9 ± 0.6
Iron	5.3 ± 0.7	4.2 ± 0.6	6.8 ± 0.9	5.2 ± 0.8	3.6 ± 0.5	2.6 ± 0.3
Magnesium	59.5 ± 2.3	53.3 ± 2.3	67.7 ± 3.7	57.6 ± 3.6	54.2 ± 4.0	42.5 ± 1.5 *
Phosphorus	<1	<1	1.3 ± 0.8	2.5 ± 0.7	<1	<1
Selenium	<1	<1	<1	1.1 ± 0.6	<1	<1
Zinc	13.3 ± 2.1	17.5 ± 3.0	23.4 ± 3.9	24.1 ± 4.4	12.8 ± 1.9	10.7 ± 1.5
Vitamin A	58.1 ± 2.2	43.1 ± 3.8 *	68.1 ± 4.0	47.6 ± 4.1 *	47.4 ± 2.4	31.9 ± 2.4 *
Thiamin	5.8 ± 1.8	8.0 ± 1.8	8.7 ± 2.6	9.4 ± 2.6	3.4 ± 1.2	4.3 ± 1.0
Riboflavin	3.2 ± 0.9	4.4 ± 1.1	7.0 ± 2.1	4.6 ± 1.5	2.4 ± 0.6	1.1 ± 0.3
Niacin	<1	1.8 ± 0.9	1.2 ± 0.9	3.6 ± 1.4	<1	<1
Folate DFE	8.3 ± 3.4	13.8 ± 1.9	17.6 ± 2.8	16.9 ± 3.3	8.8 ± 1.3	7.4 ± 1.2
Vitamin B_6_	8.5 ± 17	13.7 ± 2.3	17.7 ± 3.2	18.8 ± 3.3	6.9 ± 1.5	7.9 ± 1.0
Vitamin B_12_	1.6 ± 1.0	4.7 ± 1.8	5.3 ± 2.2	8.0 ± 2.6	3.6 ± 1.2	3.3 ± 0.7
Vitamin C	49.2 ± 3.0	48.4 ± 2.7	53.6 ± 4.7	43.3 ± 4.9	52.2 ± 1.8	35.0 ± 1.9 *
Vitamin D	97.9 ± 1.2	92.6 ± 2.0	97.1 ± 1.1	96.8 ± 1.3	94.5 ± 1.0	94.3 ± 0.9
Vitamin E	89.8 ± 1.8	92.4 ± 18	95.5 ± 1.5	85.5 ± 3.0	85.1 ± 1.7	80.2 ± 1.6
Nutrients with AI, percentage above AI						
Vitamin K	26.1 ± 3.2	32.3 ± 4.0	10.4 ± 9.8	39.6 ± 4.2 *	37.2 ± 2.8	54.1 ± 2.6 *
Choline	6.6 ± 1.2	6.1 ± 1.3	7.7 ± 2.1	6.5 ± 2.7	9.0 ± 1.5	7.8 ± 1.0

* Significant difference for consumer and non-consumer within PIR subgroups at *p* < 0.01.

**Table 3:** Usual intake of nutrients from foods and foods + dietary supplements among adults (19+ years old) by Poverty-Income-Ratio (PIR). NHANES 2009–2012, gender combined data.

Nutrients	PIR < 1.35 (*n* = 3589)		PIR 1.35 to ≤1.85 (*n* = 1184)		PIR > 1.85 (*n* = 5012)	
	Food Only	Food + Supplement	Food Only	Food + Supplement	Food Only	Food + Supplement
Nutrients with EAR						
Calcium (mg)	999 ± 17	1091 ± 18 *	939 ± 20	1059 ± 21 *	1026 ± 12	1219 ± 15 *
Copper (mg)	1.22 ± 0.01	1.39 ± 0.03 *	1.22 ± 0.02	1.45 ± 0.04 *	1.38 ± 0.02	1.76 ± 0.02 *
Iron (mg)	15.3 ± 0.2	17.6 ± 0.3 *	14.4 ± 0.3	17.2 ± 0.5 *	15.9 ± 0.1	19.2 ± 0.2 *
Magnesium (mg)	294 ± 4	307 ± 5	286 ± 6	307 ± 6	323 ± 3	358 ± 4 *
Phosphorus (mg)	1400 ± 15	1403 ± 19	1330 ± 20	1333 ± 23	1450 ± 13	1460 ± 13
Selenium (µg)	114 ± 1	122 ± 2 *	108 ± 2	120 ± 3 *	116 ± 1	140 ± 4 *
Zinc (mg)	11.6 ± 0.1	13.7 ± 0.2 *	11.0 ± 0.2	14.2 ± 0.5 *	12.1 ± 0.1	16.9 ± 0.3 *
Vitamin A (µg)	581 ± 14	768 ± 16 *	556 ± 17	824 ± 47 *	697 ± 17	1086 ± 19 *
Thiamin (mg)	1.63 ± 0.02	3.78 ± 0.58 *	1.55 ± 0.03	3.85 ± 0.65 *	1.69 ± 0.02	6.46 ± 0.63 *
Riboflavin (mg)	2.06 ± 0.03	3.19 ± 0.15 *	1.99 ± 0.04	3.35 ± 0.33 *	2.26 ± 0.03	5.67 ± 0.44 *
Niacin (mg)	25.9 ± 0.4	31.0 ± 0.8 *	24.3 ± 0.5	29.2 ± 0.7 *	26.7 ± 0.2	38.7 ± 0.9 *
Folate DFE (µg)	544 ± 9	664 ± 12 *	512 ± 10	681 ± 19 *	576 ± 7	825 ± 9 *
Vitamin B_6_ (mg)	2.08 ± 0.04	3.72 ± 0.22 *	1.93 ± 0.04	4.20 ± 0.46 *	2.21 ± 0.03	6.34 ± 0.27 *
Vitamin B_12_ (µg)	5.17 ± 0.12	35.7 ± 3.8 *	4.83 ± 0.15	41.7 ± 7.2 *	5.54 ± 0.10	58.9 ± 5.4 *
Vitamin C (mg)	81.2 ± 3.0	119 ± 4 *	83.8 ± 6.2	140 ± 14 *	87.3 ± 1.8	188 ± 6 *
Vitamin D (µg)	4.85 ± 0.14	9.45 ± 0.50 *	4.30 ± 0.14	10.2 ± 0.6 *	4.95 ± 0.11	16.4 ± 0.9 *
Vitamin E (mg)	7.76 ± 0.19	19.0 ± 1.7 *	7.49 ± 0.20	21.7 ± 2.2 *	9.03 ± 0.12	29.5 ± 1.2 *
Nutrients with AI						
Vitamin K(µg)	88.5 ± 4.0	92.0 ± 3.6	92.7 ± 4.3	97.4 ± 4.3	117 ± 3	126 ± 3
Choline (mg)	331 ± 5	331 ± 5	320 ± 7	321 ± 6	345 ± 3	346 ± 4

* Significantly different from Food Only column at *p* < 0.01.

**Table 4:** Percent of adult (19+ years old) population below Estimated Average Requirement (EAR) or above Adequate Intake (AI) of nutrients from foods and foods + dietary supplements by Poverty-Income-Ratio (PIR). NHANES 2009–2012 gender combined data.

Nutrients	PIR < 1.35 (*n* = 3589)		PIR 1.35 to ≤1.85 (*n* = 1184)		PIR > 1.85 (*n* = 5012)	
	Food Only	Food + Supplement	Food Only	Food + Supplement	Food Only	Food + Supplement
Nutrients with EAR, percentage below EAR						
Calcium	39.8 ± 1.5	33.1 ± 1.3 *^,a^	48.3 ± 2.2	38.7 ± 1.9 *^,a^	37.4 ± 1.2	24.9 ± 1.0 *^,b^
Copper	7.6 ± 0.8	6.7 ± 0.7^a^	8.9 ± 1.6	7.6 ± 1.3 ^a^	2.9 ± 0.5	2.2 ± 0.4 ^b^
Iron	5.1 ± 0.4	4.3 ± 0.4 ^a^	6.2 ± 0.7	5.3 ± 0.5 ^a^	3.0 ± 0.2	2.2 ± 0.2 *^,a^
Magnesium	56.6 ± 1.7	52.5 ± 1.8 ^a^	62.3 ± 2.3	56.2 ± 2.2 ^a^	46.9 ± 1.2	38.2 ± 1.0 *^,a^
Phosphorus	<1	<1	1.9 ± 0.6	1.9 ± 0.7	<1	<1
Selenium	<1	<1	<1	<1	<1	<1
Zinc	14.9 ± 1.7	12.3 ± 1.5 ^a^	24.0 ± 3.1	19.2 ± 1.7 ^b^	11.3 ± 1.1	7.8 ± 0.8 *^,c^
Vitamin A	52.0 ± 2.1	44.3 ± 1.6 *^,a^	57.7 ± 2.4	47.2 ± 2.5 *^,a^	37.7 ± 2.0	26.7 ± 1.2 *^,b^
Thiamin	6.8 ± 1.3	5.7 ± 1.3 ^a,b^	9.3 ± 2.0	7.5 ± 1.4 ^a^	4.1 ± 0.7	2.6 ± 0.4 ^b^
Riboflavin	3.8 ± 0.6	3.26 ± 0.46 ^a^	6.2 ± 1.1	5.4 ± 1.0 ^a^	1.5 ± 0.3	1.1 ± 0.2 ^b^
Niacin	1.2 ± 0.4	<1	2.3 ± 1.1	1.8 ± 0.9	0.8 ± 0.3	0.5 ± 0.1
Folate DFE	11.0 ± 1.8	9.0 ± 1.62 ^b^	17.2 ± 2.7	13.5 ± 1.9 ^a^	7.9 ± 0.8	5.0 ± 0.5 *^,b^
Vitamin B_6_	10.6 ± 1.2	8.2 ± 1.1 ^a^	18.4 ± 2.7	14.5 ± 2.1 ^b^	7.6 ± 0.8	4.9 ± 0.50 *^,c^
Vitamin B_12_	2.8 ± 0.9	1.9 ± 0.7	6.6 ± 1.9	5.0 ± 1.2	3.4 ± 0.8	2.1 ± 0.5
Vitamin C	48.9 ± 0.16	40.3 ± 1.8 *^,a^	49.6 ± 3.5	38.7 ± 3.6 ^a^	41.6 ± 1.6	28.4 ± 1.2 *^,b^
Vitamin D	95.5 ± 1.1	77.1 ± 1.2 *^,a^	97.0 ± 0.9	70.5 ± 2.1 *^,b^	94.2 ± 0.7	57.6 ± 0.9 *^,c^
Vitamin E	90.8 ± 1.8	74.3 ± 1.7 *^,a^	90.5 ± 2.0	69.8 ± 2.1 *^,a^	82.0 ± 1.2	52.8 ± 1.0 *^,b^
Nutrients with AI, percentage above AI						
Vitamin K	29.4 ± 3.5	32.4 ± 3.0 ^a^	32.9 ± 3.0	36.6 ± 2.7 ^a^	47.9 ± 1.8	53.5 ± 1.8 ^b^
Choline	6.7 ± 0.8	6.8 ± 0.8	7.5 ± 1.9	7.5 ± 1.8	8.3 ± 0.8	8.6 ± 0.8

* Significantly different from Food Only at *p* < 0.01; ^a–c^ Values with different letters in a row are significantly different at *p* < 0.01.

**Table 5:** Percent adult (19+ years old) population exceeding Tolerable Upper Limit of intake (UL) of nutrients from foods and foods + dietary supplements by Poverty-Income-Ratio (PIR). NHANES 2009–2012 gender combined data.

Nutrients	PIR < 1.35 (*n* = 3589)		PIR 1.35 to ≤ 1.85 (*n* = 1184)		PIR > 1.85 (*n* = 5012)	
	Food Only	Food + Supplement	Food Only	Food + Supplement	Food Only	Food + Supplement
Calcium	<1	1.69 ± 0.32 *	<1	1.89 ± 0.52	<1	4.65 ± 0.48 *
Copper	<1	<1	<1	<1	<1	<1
Iron	<1	1.36 ± 0.17 *	<1	1.72 ± 0.51 *	<1	1.81 ± 0.18 *
Phosphorus	<1	<1	<1	<1	<1	<1
Selenium	<1	<1	<1	<1	<1	<1
Zinc	<1	<1	<1	2.17 ± 0.63 *	<1	2.49 ± 0.33 *
Vitamin A	<1	<1	<1	<1	<1	<1
Niacin	ND	ND	ND	ND	ND	ND
Folate DFE	<1	<1	<1	1.26 ± 0.32 *	<1	2.18 ± 0.20 *
Vitamin B_6_	<1	<1	<1	<1	<1	1.06 ± 0.21 *
Vitamin C	<1	<1	<1	<1	<1	<1
Vitamin D	<1	<1	<1	<1	<1	1.55 ± 0.41 *
Vitamin E	<1	<1	<1	<1	<1	<1
Choline	<1	<1	<1	<1	<1	<1

* Significantly different from Food Only at *p* < 0.01. Vitamin A, folate, and vitamin E ULs based on retinol, folic acid, and added alpha tocopherol, respectively. ND: Not determined as niacin UL is based on a particular form of niacin (nicotinic acid) which is not quantified in NHANES.
